# On the Use and Abuse of Alcoholic Liquors, in Health and Disease

**Published:** 1850-11

**Authors:** 


### ARTICLE VI.

*On the use and abuse of Alcoholic Liquors, in Health and Disease.*---Prize Essay, by WILLIAM B. CARPENTER, M.D., F. R.S., F.G.S., Examiner in Physiology in the University of London, Professor of Medical Jurisprudence in University College, and author of "Principles of Human Physiology," etc., etc. Philadelphia: Lea & Blanchard, 1850; pp. 201; 12mo. (From the Publishers, and for sale by J. Keen, Jr. & Bro.)

To this little volume was adjudged the prize of a hundred guineas for the best essay on the use and abuse of Alcoholic Liquors, as a beverage or as Therapeutic agents. Intemperance is a subject of extreme interest to medical men, as bearing on public Hygiene; and we are glad to see a man occupying the eminent position of Professor Carpenter devoting his attention to it. We cannot give to our readers more fully the design and scope of the volume, than by presenting them the following extract from the author's preface. He desires to impress upon his readers the following conclusions.

"In the *first* place. That, from a scientific examination of the *modus operandi* of Alcohol upon the Human body, when taken in a *poisonous* dose, or to such an extent as to produce Intoxication, we may fairly draw inferences with regard to the specific effects which it is likely to produce, when repeatedly taken in excess, but not to an immediately fatal amount.

*Secondly*, That the consequences of the *excessive* use of Alcoholic liquors, as proved by the experience of the Medical Profession, and universally admitted by medical writers, being precisely such as the study of its effects in poisonous and immediately fatal doses would lead us to anticipate, we are further justified in expecting that the habitual use of smaller quantities of these liquors, if sufficiently prolonged, will ultimately be attended, in a large proportion of cases, with consequences prejudicial to the human system,---the morbid actions thus engendered being likely rather to be chronic, than acute, in their character.

"*Thirdly*, That as such morbid actions are actually found to be among the most common disorders of persons advanced in life, who have been in the habit of taking a 'moderate' allowance of alcoholic liquors, there is very strong ground for regarding them as in a great degree dependent upon the asserted cause; although the long postponement of their effects may render it impossible to *demonstrate* the existence of such a connection.

"*Fourthly*, That the preceding conclusion is fully borne out by the proved results of the 'moderate' use of Alcoholic liquors, in producing a marked liability to the acute forms of similar diseases in hot climates, where their action is accelerated by other conditions; and also by the analogous facts now universally admitted, in regard to the remotely injurious effects of slight excess in diet, imperfect aeration of the blood, insufficient repose, and other like violations of the Laws of Health, when habitually practiced through a long period of time.

"*Fifthly*, That the capacity of the healthy human system to sustain as much bodily or mental labor as it can be legitimately called upon to perform, and its power of resisting the extremes of Heat and Cold, as well as other depressing agencies, are not augmented by the use of Alcoholic liquors; but that, on the other hand, their use, under such circumstances, tends positively to the impairment of that capacity.

"*Sixthly*, That, where there is a deficiency of power, on the part of the system, to carry on its normal actions with the energy and regularity which constitute health, such power can rarely be imparted by the habitual use of Alcoholic liquors; its deficiency being generally consequent upon some habitual departure from the laws, for which the use of Alcoholic liquors cannot compensate; and the employment of such liquors, although with temporary effect of palliating the disorder, having not merely a remotely injurious effect *per se*, but also tending to mask the action of other morbific causes, by rendering the system more tolerant of them.

"*Seventhly*, That, consequently, it is the duty of the Medical Practitioner to discourage as much as possible the *habitual* use of Alcoholic liquors, in however 'moderate' a quantity, by all persons in ordinary health; and to seek to remedy these slight departures from health which result from the 'wear and tear' of active life by the means which shall most directly remove or antagonize their causes, instead of by such as simply palliate their effects.

"*Eighthly*, That, whilst the habitual use of Alcoholic liquors, even in the most 'moderate' amount, is likely (except in a few rare instances) to be rather injurious than beneficial, great benefit may be derived, in the treatment of disease, from the *medicinal* use of Alcohol in appropriate cases; but that the same care should be employed in the discriminating selection of those cases, as would be taken by the conscientious practitioner in regard to the administration of any other powerful remedy which is poisonous in large doses."

The following powerful paragraphs contain our author's views on the *Total Abstinence* question:

"He cannot allow it to go forth, however, without expressing his conviction that, whilst there are adequate Medical reasons for Abstinence from the *habitual* use of even a 'moderate' quantity of Alcoholic liquors, there are also strong Moral grounds for Abstinence from that *occasional* use of them which is too frequently thought to be requisite for social enjoyment, and to form an essential part of the rites of hospitality. The experience of every practitioner must bring the terrible results of Intemperance frequently before his eyes; but, whilst he is thus rendered familiar with its consequences as regards *individuals*, few, save those who have expressly inquired into the subject, have any idea of the extent of the *social* evils resulting from it, or of the degree in which they press upon every member of the community. The Author believes that he is fully justified in the assertion that, among those who *have* thus inquired, there is but one opinion as to the fact that, of all the causes which are at present conspiring to degrade the physical, moral, and intellectual condition of the mass of the people, there is not one to be compared in potency with the *Abuse of Alcoholic liquors;* and that, if this could be done away with, the removal of all the other causes would be immeasurably promoted. Every one who wishes well to his kind, therefore, must be interested in the inquiry how this monster-evil can be best eradicated.

"Now the Author considers that the best answer to this inquiry has been found in the results of experience. A fair trial has been given, both in this country and the United States, to societies which advocated the principle of *Temperance*, and which enlisted in their support a large number of intelligent and influential men; but it has been found that little or no good has been effected by them, among the classes on whom it was most desirable that their influence should be exerted, except where those who were induced to join them really adopted the *Total Abstinence* principle. Though he agrees fully with those who maintain that, *if* all the world would be *really temperate*, there would be no need of Total Abstinence Societies, the Author cannot adopt the inference that those who desire to promote the Temperance cause may legitimately rest satisfied with this measure of advocacy. For sad experience has shown that a large proportion of mankind *cannot*, partly for want of the self-restraint which proceeds from moral and religious culture, be temperate in the use of Alcoholic liquors; and that the reformation of those who have acquired habits of intemperance *cannot* be accomplished by any means short of entire Abstinence from fermented liquors. Further, experience has shown that, in the present dearth of effectual education among the masses, and with the existing temptations to Intemperance arising out of the force of example, the almost compulsory drinking-usages of numerous trades, and the encouragement which in various ways is given to the abuse of Alcoholic liquors, nothing short of Total Abstinence can prevent the continuance, in the rising generation, of the terrible evils which we have at present to deplore. And lastly, experience has also proved that this reformation cannot be carried to its required extent, without the co-operation of the educated classes; and that their influence can only be effectually exerted by *example*. There is no case in which the superiority of example over mere precept is more decided and obvious than it is in this. 'I practice total abstinence myself,' is found to be worth a thousand exhortations; and the lamentable failure of the advocates who cannot employ this argument should lead all those whose position calls upon them to exert their influence to a serious consideration of the claims which their duty to society should set up, in opposition to their individual feelings of taste or comfort."

How many of our readers acknowledge the force of these remarks, and are yet compelled to say with the Roman Poet,

> ——— Video, proboque meliora,
> Deteriora sequor?

We ask this question without intending any invidious insinuations, but merely to induce the profession to take into serious consideration their duty in the premises as conservators of the public health, and as good citizens solicitous to remove a festering spot which has long been threatening to destroy the very vitals of the body politic and social.

The high professional reputation of Prof. Carpenter is a sufficient guaranty that the subject is treated ably; and his distinct avowal that he is a member of no special Temperance organization is strong presumptive proof that he has performed his task impartially. We therefore recommend this volume as the best treatise extant on the subject of the use and abuse of Alcoholic liquors. M.

